# Comparison of simplicity, convenience, safety, and cost-effectiveness between use of insulin pen devices and disposable plastic syringes by patients with type 2 diabetes mellitus: a cross-sectional study from Bangladesh

**DOI:** 10.1186/s12902-023-01292-8

**Published:** 2023-02-13

**Authors:** A. B. M. Kamrul-Hasan, Mohammad Abdul Hannan, Muhammad Shah Alam, Mohammad Motiur Rahman, Md. Asaduzzaman, Marufa Mustari, Ajit Kumar Paul, Md. Lutful Kabir, Sumon Rahman Chowdhury, Samir Kumar Talukder, Sourav Sarkar, Muhammad Abdul Hannan, Md. Rashedul Islam, Mohammad Hasan Iftekhar, Md. Abdul Bari Robel, Shahjada Selim

**Affiliations:** 1 Department of Endocrinology, Mymensingh Medical College, Mymensingh, Bangladesh; 2 Department of Endocrinology, North East Medical College, Sylhet, Bangladesh; 3 Department of Medicine, Army Medical College, Cumilla, Bangladesh; 4 Department of Medicine, Rajshahi Medical College Hospital, Rajshahi, Bangladesh; 5 Department of Endocrinology, Shaheed Sheikh Abu Naser Specialized Hospital, Khulna, Bangladesh; 6 Department of Endocrinology, Bangabandhu Sheikh Mujib Medical University, Dhaka, Bangladesh; 7 Department of Endocrinology, Mainamoti Medical College, Cumilla, Bangladesh; 8 Department of Endocrinology, Rangpur Medical College, Rangpur, Bangladesh; 9 Department of Diabetes, Endocrinology and Metabolism, Chittagong Diabetic General Hospital, Chattogram, Bangladesh; 10 Department of Medicine, Boalkhali Upazila Health Complex, Chattogram, Bangladesh; 11 Department of Medicine, 250-Bedded General Hospital, Sherpur, Bangladesh; 12 Department of Neurology, BIRDEM General Hospital, Dhaka, Bangladesh; 13 Department of Biochemistry, Abdul Malek Ukil Medical College, Noakhali, Bangladesh; 14 Department of Endocrinology, Cumilla Medical College, Cumilla, Bangladesh

**Keywords:** Insulin pen, Insulin syringes, Simplicity, Convenience, Safety, Cost-effectiveness

## Abstract

**Introduction:**

Insulin pen devices and disposable plastic insulin syringes are two common tools for insulin administration. This study aims to compare the simplicity, convenience, safety, and cost-effectiveness of insulin pens versus syringe devices in patients with type 2 diabetes mellitus (T2DM).

**Methods:**

A cross-sectional study was conducted at 14 diabetes clinics throughout Bangladesh from November 2021 to April 2022 among adults with T2DM injecting insulin by pen devices or disposable insulin syringes at least once a day for at least one year by purposive sampling. The simplicity, convenience, and safety of insulin devices were assessed using a structured questionnaire, and the study subjects were scored based on their answers; higher scores indicated a poorer response. Total scores for simplicity, convenience, and safety were obtained by adding the scores for relevant components. Their average monthly medical expense and cost of insulin therapy were recorded. The median values of the total scores and monthly expenses were compared between pen devices and disposable syringe users.

**Results:**

737 subjects were evaluated; 406 were pen users, and 331 were vial syringe users. The pen users had lower median scores for simplicity [6.0 (5.0–8.0) vs. 7.0 (5.0–9.0), *p* = 0.002], convenience [4.0 (3.0–6.0) vs. 5.0 (4.0–6.0), *p* < 0.001], and safety [7.0 (6.0–8.0) vs. 7.0 (6.0–9.0), *p* = 0.008] than vial syringe users. Pen devices were more expensive than vial syringes in terms of average medical expense per month [BDT 5000 (3500–7000) vs. 3000 (2000–5000), *p* < 0.001], the total cost of insulin therapy per month [BDT 2000 (1500–3000) vs. 1200 (800–1700), *p* < 0.001] and cost per unit of insulin used [BDT 2.08 (1.39–2.78) vs. 0.96 (0.64–1.39), *p* < 0.001]. Non-significant differences in favor of pens were observed in HbA1c levels [8.7 (7.8–10) vs. 8.9 (7.9–10)%, *p* = 0.607] and proportions of subjects having HbA1c < 7% (6.9 vs. 6.3%, *p* = 0.991).

**Conclusion:**

Insulin pens are simpler, more convenient, and safe but more expensive than vial syringes. Glycemic control is comparable between pen and syringe users. Long-term follow-up studies are needed to determine the clinical and economic impacts of such benefits of insulin pens.

## Introduction

Diabetes mellitus (DM) is a worldwide epidemic that requires continuous long-term medical care. Type 2 diabetes mellitus (T2DM) is the most common type of diabetes, accounting for over 90% of all diabetes worldwide. Currently, more than 13.1 million adults in Bangladesh have diabetes, and the prevalence is increasing [1]. Absolute deficiency of endogenous insulin secretion makes exogenous insulin an inevitable option for type 1 DM. In the long run, patients with T2DM also require exogenous insulin regularly due to progressive beta-cell failure and subsequent ineffectiveness of other glucose-lowering drugs [2]. The discovery of insulin revolutionized diabetes management and helped patients achieve better glycemic control and reduce the incidence of micro-and macrovascular complications [3]. Traditionally, insulin is administered by insulin syringes. During the last few decades, there has been a continuum of advancements in the insulin delivery system, including the introduction of insulin pen devices, insulin pumps, inhalational insulin, etc. [4]. Despite this advancement in insulin delivery devices, vial syringes, and pens remain the mainstay of insulin use in this area [5, 6]. For injecting nature and associated hazards, insulin injection is seldom welcomed by the patients, and most patients experience difficulty in injecting insulin [7, 8]. The negative impacts of insulin injection led to treatment nonadherence and may be barriers to achieving good glycemic control [9]. Evidence suggests that insulin pens offer convenience, less pain, and better treatment adherence and health outcomes than traditional vial syringes. However, pen devices are more expensive and less affordable than insulin vials, especially in low- and middle-income countries [4]. Furthermore, some patients may find pens more challenging to operate than vial syringes [10].

Although several observational studies and randomized control trials have compared the various aspects of the use of insulin pens and syringe vials, [11–18] those involved small samples; moreover, there is no study from Bangladesh. The present study aimed to compare the simplicity, convenience, safety, and cost-effectiveness of insulin pen devices versus conventional insulin vial syringes.

## Materials and methods

### Ethical considerations

The institutional review board of Mymensingh Medical College approved the study protocol (MMC/IRB/2021/417, Date: 15 October 2021). Informed written consent was taken from the study participants. The study was conducted according to Good Clinical Practice and the Helsinki accords. Participants’ identities were kept confidential at all times. Subjects were neither placed at any health risk by the study nor by treatment decisions based on it. In addition, no financial compensation was offered for participation.

### Study design, setting, and participants

We conducted this cross-sectional study at 14 diabetes outpatient clinics throughout Bangladesh from November 2021 to April 2022. Insulin-treated, self-injecting adults (≥ 18 years) with T2DM who had been injecting insulin by pen devices or disposable insulin syringes at least once a day for at least one year and consented to the study were included in the study by purposive sampling. The participants were divided into two groups- the first group consisted of study subjects injecting insulin with a pen device, and the second group consisted of subjects injecting insulin with a disposable insulin syringe. Subjects with other types of diabetes, pregnant and postpartum women taking insulin, insulin pump users, those injected by others (family members or health-care professionals), those diagnosed with severe psychiatric illness, those with acute illness or with recent (within six months) severe complications of diabetes like vascular events, those with debilitating chronic complications of diabetes or comorbidities, and those lacking relevant data required for the study, were excluded.

### Questionnaire development

A structured questionnaire, designed and used among patients with T2DM in India by Singh et al. for a similar study, [11] with some modifications made by the investigators in response to a pilot study using the Bangla version of the questionnaire among twenty (ten in each group) subjects, was used for data collection. The study questionnaire is divided into four segments. The first segment consists of general questions about the subjects’ demographics and anthropometric characteristics as well as the duration of diabetes, the duration of insulin use, the type of insulin used, the total daily dose of insulin, the number of times blood glucose was self-monitored, the presence of comorbidities, diabetic complications and lipodystrophy, glycated hemoglobin (HbA1c) within previous one month in their medical records. The segment also enquires about the cost of treatment by asking each subject about the average medical expense per month and their current monthly cost of insulin therapy (insulin, disposables, and cleaning equipment). The second segment consists of five questions on simplicity (Table 1), which was assessed by asking what the total number of missed doses was in the five days preceding the interview, the ease of injecting insulin and handling the device during calibration of the dose, the ease of changing needles, and the ease of insulin storage. The third segment has three questions on convenience (Table 2), which was assessed by asking the total number of steps and the time taken to administer insulin. Additionally, the questionnaire inquired about the ease of administering insulin on trips and events/meals outside the subject’s home. The fourth segment consists of five questions on safety (Table 3). Safety was assessed by asking how painful the process was of injecting the insulin, the number of bruising episodes noted in the five days preceding the interview, the number of self-reported episodes of hyperglycemia in the week before the interview, the number of hypoglycemic episodes during the last three months preceding the interview and recollection of the number of insulin cartridges/vials accidentally broken in the 12 months preceding the interview.

**Table 1:** Questionnaire for assessing the simplicity of insulin delivery device

Simplicity assessment questions			
Sl. No.	Question	Options	Score*
1	How many of doses of insulin have you missed in the last 5 days?	None	1
		1–3 doses	2
		> 3 doses	3
2	How easy is to inject your insulin?	Easy	1
		Intermediate	2
		Hard	3
3	How easy is it to calibrate the dose of your insulin?	Easy to learn	1
		Acceptable	2
		Hard	3
4	How easy is it to change needles (removing/connecting)?	Easy to learn	1
		Acceptable	2
		Hard	3
5	How easy is it to store your insulin?	Easy to learn	1
		Acceptable	2
		Hard	3

*Scoring as per response. A minimum score of 5 and a maximum score of 15. A lower score indicates a simpler device, and a higher score denotes a more complicated device

**Table 2:** Questionnaire for assessing the convenience of insulin delivery device

Convenience assessment questions			
Sl. No.	Question	Options	Score*
1	How much time do you spend in injecting insulin once the insulin is at room temperature?	< 1 min	1
		1–2 min	2
		> 2 min	3
2	How many steps you have to remember to take when you inject your insulin?	< 2	1
		2–4	2
		> 4	3
3	How easy is it to carry insulin on holidays and for meals outside the home?	Easy	1
		Acceptable	2
		Hard	3

*Scoring as per response. A minimum score of 3 and a maximum score of 9. A lower score indicates a more convenient device, and a higher score indicates an inconvenient device

**Table 3:** Questionnaire for assessing the safety of insulin delivery device

Safety assessment questions			
Sl. No.	Question	Options	Score*
1	How painful is the process of injecting your insulin?	Acceptable pain	1
		Bearable pain	2
		Unbearable pain	3
2	How many bruising episodes at injection sites have you had in the last 5 days?	None	1
		1 episode	2
		> 1 episode	3
3	How many episodes of high sugars (your perception) have you noticed in the last one week?	None	1
		1 episode	2
		> 1 episode	3
4	How many episodes of low sugars (your perception) have you noticed in the last one week?	None	1
		1 episode	2
		> 1 episode	3
5	How many times have your broken your insulin vial/cartridge in the past one year?	None	1
		1 episode	2
		> 1 episode	3

*Scoring as per response. A minimum score of 5 and a maximum score of 15. A lower score indicates a safer device, and a higher score denotes a more unsafe device

Each study subject was interviewed separately by the corresponding investigator, who filled in the questionnaire based on the answers given by the subject. A score was given for each answer. The highest obtainable scores for simplicity, convenience, and safety were 15, 9, and 15, respectively. The scoring system was designed to place the responses in descending order, such that higher scores indicated a poorer response.

### Statistical analysis

Data were analyzed using the IBM SPSS Statistics for Macintosh, Version 28.0 (IBM Corp. Released 2021, Armonk, NY) software. Kolmogorov–Smirnov test was used to test the normality of data; the continuous variables with normal distribution and without a normal distribution were expressed as mean ± standard deviation (SD) and median (interquartile range, IQR), respectively. The categorical variables were presented as the percentage (numbers). Student’s *t*-test, Chi-square test, Independent-Samples Median test, and Mann-Whitney-U test were performed to compare the variables between different groups as appropriate. A two-sided *p* value of less than 0.05 indicates statistical significance.

## Results

### Characteristics of the participants

A total of 737 subjects with T2DM injecting insulin at least once a day for at least one year were evaluated in this study; 406 were pen users, and 331 were vial syringe users. Characteristics of the participants are summarized in Table 4. Subjects in the two groups were similar in age, male: female ratio, body mass index (BMI), duration of diabetes, duration of insulin use, the total daily dose of insulin, presence of comorbidities, diabetic complications, and lipodystrophy. HbA1c levels were also similar in them. More people among insulin pen users were from urban than syringe users. The basal-only regimen was more frequently used among the pen users, and premixed insulin was more frequent among the syringe users. A higher number of the pen users owned glucometers and did self-monitoring of blood glucose than the syringe users.

**Table 4:** Baseline characteristics of the participants

Variables	All subjects *(N = 737)*	Pen users *(n = 406)*	Vial syringe users *(n = 331)*	*p* value
	median (IQR) or n (%)			
Age (years)	52 (45–60)	52 (45–60)	51 (45–60)	0.161
Male: Female Ratio	312:425	169:237	143:188	0.708
Residence:				
Urban	405 (55.0%)	278 (68.5%)	127 (38.4%)	< 0.001
Sub-urban	177 (24.0%)	80 (19.7%)	97 (29.3%)	
Rural	155 (21.0%)	48 (11.8%)	107 (32.3%)	
BMI (kg/m^2^)	26.3 (24.5–28.6)	26.2 (24.6–28.4)	26.5 (24.1–28.7)	0.448
Duration of DM (years)	10 (6–15)	10 (6–15)	10 (6–15)	0.508
Duration of insulin use (years)	3 (2–7)	3 (2–8)	4 (2–7)	0.692
Total daily dose of insulin (Units)	36 (28–50)	36 (24–50)	36 (30–52)	0.676
Insulin regimen:				
Basal only	111 (15.1%)	91 (22.4%)	20 (6.0%)	< 0.001
Basal-Plus	8 (1.1%)	5 (1.2%)	3 (0.9%)	
Premixed	452 (61.3%)	226 (55.7%)	226 (68.3%)	
Basal-Bolus	123 (16.7%)	65 (16.0%)	58 (17.5%)	
Bolus only	43 (5.8%)	19 (4.7%)	24 (7.3%)	
Own glucometer	555 (75.3%)	349 (86.0%)	206 (62.0%)	< 0.001
SMBG frequency:				
Once/day	34 (4.6%)	25 (6.2%)	9 (2.7%)	< 0.001
Twice /day	22 (3.0%)	12 (3.0%)	10 (3.0%)	
Thrice or more/day	87 (11.8%)	74 (18.2%)	13 (3.9%)	
Not daily but at least once/week	243 (33.0%)	142 (35.0%	101 (30.5%)	
Less than weekly	164 (22.3%)	94 (23.2%)	70 (21.1%)	
Never do SMBG	187 (25.4%)	59 (14.5%)	128 (38.7%)	
Comorbidity present	610 (82.8%)	336 (82.8%)	274 (82.8%)	1.000
Diabetic complication present	488 (66.2%)	271 (66.7%)	217 (65.6%)	0.754
Lipodystrophy	112 (15.2%)	61 (15.0%)	51 (15.4%)	0.918
HbA1c (%)	8.8 (7.9–10)	8.7 (7.8–10)	8.9 (7.9–10)	0.607

*BMI* Body mass index, *DM* Diabetes mellitus, *SMBG* Self-monitoring of blood glucose

**Table 5:** Comparison of responses to the questions for assessment of simplicity of insulin delivery devices (pens vs. vial syringe users)

Question no.	Question	Response	Pen users *(n = 406)*	Vial syringe users *(n = 331)*	*p* value
1	How many of doses of insulin have you missed in the last 5 days?	None	77.8%	65.6%	< 0.001
		1–3 doses	19.7%	28.4%	
		> 3 doses	2.5%	6.0%	
2	How easy is to inject your insulin?	Easy	64.0%	55.0%	0.039
		Intermediate	25.4%	30.5%	
		Hard	10.6%	14.5%	
3	How easy is it to calibrate the dose of your insulin?	Easy to learn	71.2%	53.2%	< 0.001
		Acceptable	21.2%	31.7%	
		Hard	7.6%	15.1%	
4	How easy is it to change needles (removing/connecting)?	Easy	68.5%	61.6%	0.151
		Acceptable	17.7%	21.8%	
		Hard	13.8%	16.6%	
5	How easy is it to store your insulin?	Easy	83.5%	76.7%	0.044
		Acceptable	14.3%	18.7%	
		Hard	2.2%	4.5%	

% indicates the percentage of column totals

**Table 6:** Comparison of responses to the questions for assessment of convenience of insulin delivery devices (pens vs. vial syringes)

Question no.	Question	Response	Pen users *(n = 406)*	Vial syringe users *(n = 331)*	*p* value
1	How much time do you spend in injecting insulin once the insulin is at room temperature?	< 1 min	54.7%	35.3%	< 0.001
		1–2 min	35.5%	48.6%	
		> 2 min	9.9%	16.0%	
2	How many steps you have to remember to take when you inject your insulin?	< 2	53.2%	36.0%	< 0.001
		2–4	38.2%	54.7%	
		> 4	8.6%	9.4%	
3	How easy is it to carry insulin on holidays and for meals outside the home?	Easy	56.4%	39.6%	< 0.001
		Acceptable	24.4%	35.3%	
		Hard	19.2%	25.1%	

% indicates the percentage of column totals

**Table 7:** Comparison of responses to the questions for assessment of safety of insulin delivery devices (pens vs. vial syringes)

Question no.	Question	Response	Pen users *(n = 406)*	Vial syringe users *(n = 331)*	*p* value
1	How painful is the process of injecting your insulin?	Acceptable pain	74.6%	64.4%	0.007
		Bearable pain	23.9%	34.4%	
		Unbearable pain	1.5%	1.2%	
2	How many bruising episodes at injection sites have you had in the last 5 days?	None	74.9%	71.3%	0.020
		1 episode	20.4%	18.7%	
		> 1 episode	4.7%	10.0%	
3	How many episodes of high sugars (your perception) have you noticed in the last one week?	None	41.6%	33.5%	< 0.001
		1 episode	31.5%	25.4%	
		> 1 episode	26.8%	41.1%	
4	How many episodes of low sugars (your perception) have you noticed in the last one week?	None	69.7%	73.1%	0.596
		1 episode	20.2%	17.8%	
		> 1 episode	10.1%	9.1%	
5	How many times have your broken your insulin vial/cartridge in the past one year?	None	82.5%	71.9%	0.003
		1 episode	11.8%	18.4%	
		> 1 episode	5.7%	9.7%	

% indicates the percentage of column totals

**Table 8:** Comparison of total simplicity, convenience, and safety scores between pen users and vial syringe users

Variables	All subjects (N = 737)	Pen users *(n = 406)*	Vial syringe users *(n = 331)*	*p* value
Total simplicity score	6.0 (5.0–8.0)	6.0 (5.0–8.0)	7.0 (5.0–9.0)	0.002^*^ < 0.001^†^
Total convenience score	5.0 (4.0–6.0)	4.0 (3.0–6.0)	5.0 (4.0–6.0)	< 0.001^*†^
Total safety score	7.0 (6.0–8.0)	7.0 (6.0–8.0)	7.0 (6.0–9.0)	0.008^*^ < 0.001^†^

^*^p value for differences in the median across the categories

^†^*p* value for differences in the distribution of variables across the categories

### Glycemic control

**Fig. 1:**
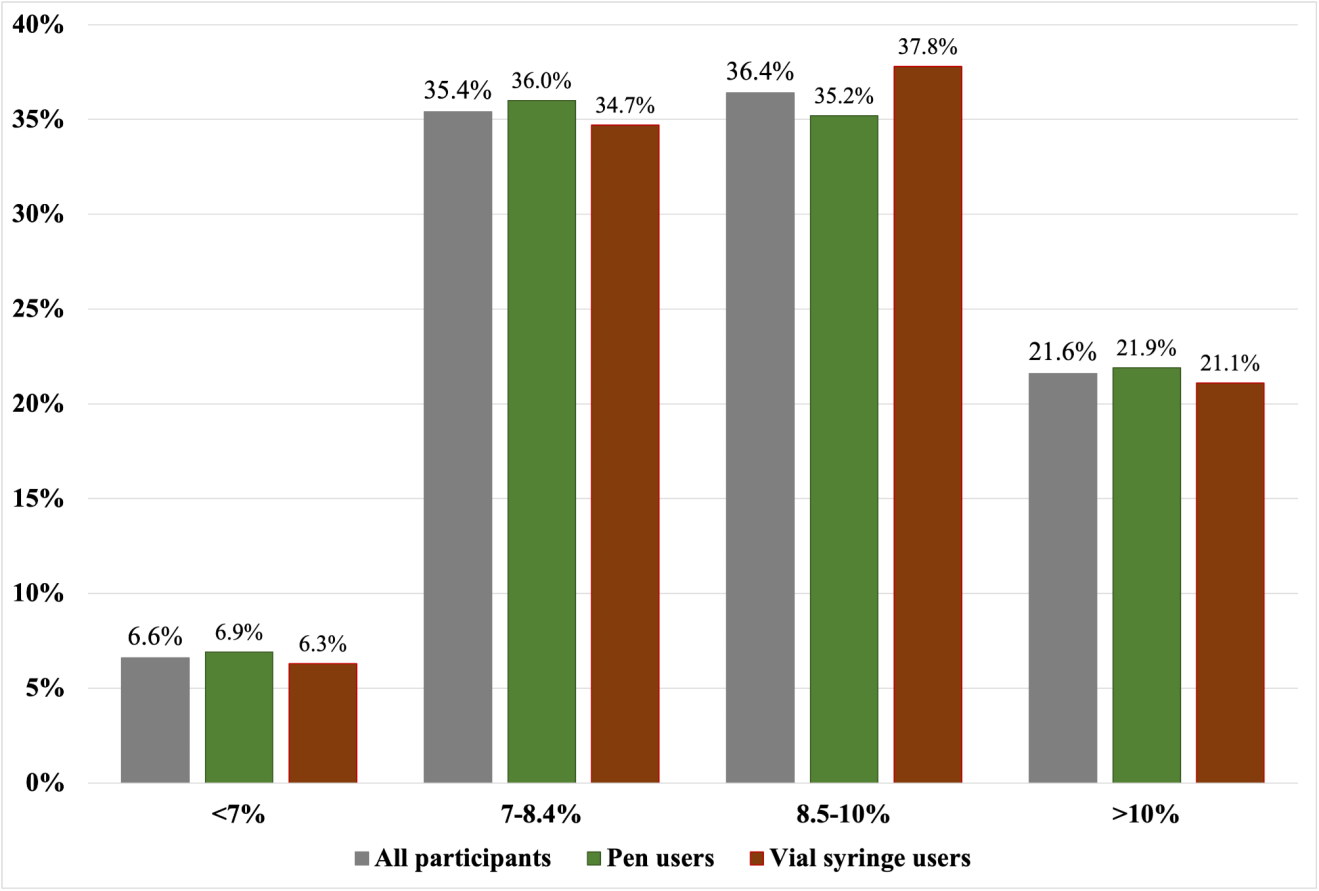
HbA1c levels in pen users versus vial syringe users

Overall, only 6.6% achieved the target HbA1c of < 7%. Pen and syringe users had similar (*p* = 0.991) proportions of subjects at different stages of glycemic control (Fig. 1).

### Comparison of simplicity, convenience, and safety of insulin delivery devices (pens vs. vial syringes)

The response of the study subjects to the questions for assessment of simplicity, convenience, and safety of insulin delivery devices (pens vs. syringe-vials) are given in Table 5, 6, and 7, respectively. The response favored the pen devices in every question except for the ease of changing needles (removing/connecting) and the number of hypoglycemic episodes in the preceding week. A comparison of total simplicity, convenience, and safety scores in pen users versus syringe users is given in Table 8. The differences in the distribution of these variables across the two groups were statistically significant (*p* < 0.001), and the medians of simplicity, convenience, and safety scores were significantly higher (*p* values are significant for all) among the pen users than the vial syringe users.

### Comparison of the treatment-related expense of insulin delivery devices (pens vs. vial syringes)

Using the pen device for insulin administration was more expensive than a disposable syringe device in terms of average medical expense per month, the total cost of insulin therapy (insulin, disposables, cleaning equipment) per month and the price per unit of insulin used (Table 9).

**Table 9:** Comparison of average medical expense per month and total cost (in BDT) of insulin therapy in pen users versus vial syringe users

Variables	All subjects (N = 737)	Pen users *(n = 406)*	Vial syringe users *(n = 331)*	*p* value
Average medical expense per month (BDT)	4000 (3000–6000)	5000 (3500–7000)	3000 (2000–5000)	< 0.001
Total cost of insulin therapy per month (BDT)	1600 (1000–2400)	2000 (1500–3000)	1200 (800–1700)	< 0.001
Cost per unit insulin used (BDT)	1.45 (0.89–2.32)	2.08 (1.39–2.78)	0.96 (0.64–1.39)	< 0.001

## Discussion

In this cross-sectional study, including 737 subjects (406 pen users, 331 syringe-vial users) with T2DM injecting insulin at least once a day for at least one year, insulin pens were simpler and more convenient to use and safe but expensive than vial syringes. Glycemic control was comparable between the two groups.

Most people with diabetes inject insulin with a syringe or pen as the insulin delivery method; other methods of insulin delivery, including insulin inhalers, insulin pumps, or automated insulin delivery devices, are seldom used. Delivering insulin through either insulin pens or syringes can safely and effectively lower blood glucose. The factors influencing the decision to choose among delivery systems include patient preferences, cost, insulin type, dosing regimen, and self-management capabilities [19]. In a meta-analysis mainly comprising the adults with T2DM, the pen devices were superior to syringe vials in terms of mean HbA1c change, hypoglycemic episodes, adherence, and persistence to insulin, although no difference was observed in the number of patients achieving glycemic target (HbA1c < 7%). There was a tendency to favor pen devices, and using pens improved the quality of life [20].

In this study, we observed that patients using the insulin pen device had fewer missed doses and found it easier to calibrate the dose, inject insulin, and store their pen device than syringe users, making pen devices simpler to use. Singh et al. had a similar observation in Indian subjects with T2DM treated with insulin injections [11]. A higher percentage of Lebanese insulin pen users (95.2%) found the method easy to use compared to insulin syringe users (46.7%) [12]. Compared with vials and syringes, pens were easier to use and operate and demonstrated superior dose accuracy in a study by Ignaut et al. [13]. Korytkowski et al. also observed that it is easier to use overall and found the insulin dose scale on the pen easier to read than the vial/syringe [14]. Insulin pens may allow people with vision impairment or dexterity issues to dose insulin accurately [21].

Pen devices were also found more convenient to carry and use outside the home, with less time and fewer steps involved in the injection process than syringes in this study; the findings are similar to Singh et al. [11]. Compared to syringes, patients found it more discreet to use pen devices in public places, and consequently, they felt greater lifestyle flexibility with pens [14, 21]. In a study, 85.7% of pen users found it more convenient to shift to pens, and 86.7% of syringe users would want to change the pen if it had the exact cost [12].

Although the number of episodes of hypoglycemia was comparable between pen and syringe-vial groups, insulin pen users reported less pain during injection, fewer incidents of bruising at injection sites, fewer hypoglycemic episodes, and fewer occasions of accidental breaking of insulin devices, making the pen safer to use. These results concord with the findings of Singh et al. [11]. The pain involved in the self-injection of insulin is partially related to the characteristics of the needle, particularly its diameter. Pen needles may be sharper and thinner than syringe needles because they do not have to penetrate the insulin vial stopper before injection [21]. Patients in several studies reported less injection pain associated with insulin pen devices than with vial syringes [12, 21]. Like us, Ahmann et al. found comparable incidences of hypoglycemia in the two groups [15]. Contrary to us, most studies reported a statistically significant difference in hypoglycemic incidences favoring pen devices [20, 22]. Not all studies reported superior safety profiles for insulin pens over syringes; similar safety profiles in the two groups are reported by Korytkowski et al. during treatment periods with basal insulin glargine [14].

Overall, glycemic control was unsatisfactory in this study; the median HbA1c was 8.8%, and only 6.6% achieved the target HbA1c of < 7%. Insulin pen and syringe users had similar HbA1c levels and proportions to subjects at different stages of glycemic control. Insulin pens generally show equivalence or minor improvements in glycemic outcomes compared to using vial syringes. Lower HbA1c levels among pen users than syringe users have been reported in some studies [11, 14, 22]. The pen group also showed a more significant reduction in HbA1c in 24 weeks of follow-up in a study by Machry et al. [16]. Ahmann et al. reported no difference between the two groups in the percentage of patients that achieved HbA1c < 7% (37.7% vs. 37%; *p* = 0.89) after a 40-week follow-up [15]. A meta-analysis showed a non-statistically significant trend toward pen devices in the percentage of patients who reached HbA1c < 7% [20]. So, it is tough to comment whether pen devices offer better glycemic control than syringe use. In addition to the use of specific insulin devices, many factors affect glycemic control, which may explain such heterogenicity of the study results.

Using pen devices for insulin administration is more expensive than disposable syringe devices in terms of average medical expense per month, the total cost of insulin therapy per month, and the cost per unit of insulin used, according to the current study. Vials are cheaper than pen cartridges. Though many insulin types are available for purchase as pens or vials, others may only be available in one form or another, and there may be cost differences between them [19, 23]. Analog insulins are costlier than human insulins and more frequently injected with pen devices which may be associated with the higher cost of pen use [24]. Prescription costs of syringes were lower, and expenses for pens were higher in patients who were switched from the syringe to pen versus those who remained on syringe therapy [25]. Other studies also identified that treatment using pen devices was costlier when compared to using syringes [11, 22]. Despite the higher prescription costs of insulin pens than vial syringes, previous studies reported similar or even lower all-cause and diabetes-related total annualized healthcare costs [17, 22, 25]. Studies indicated that insulin pen devices are associated with improved adherence and persistence with therapy instead of vial syringes. The healthcare resource utilization and costs associated with them decreased with the use of pen devices compared to vial syringes [17, 18, 21, 26]. These are behind the users’ higher preference for pens over vial syringes and more robust recommendations for pens over vial syringes by healthcare professionals [13–15, 21]. This higher preference for pens is associated with an increasing use trend, while insulin vial syringes declined in parallel [23].

The major limitation of this study is that we used a non-validated questionnaire. The questionnaire was administered by multiple investigators, which could allow for bias in the scoring. Moreover, the observed difference in the scores between the two groups still waits to be clinically translated. We analyzed the short-term (one month) cost for insulin and total treatment cost, restraining us from comparing the long-term cost-effectiveness of pens and vials-syringes. Furthermore, we did not consider the type of insulins used (i.e., human or analog, originator or biosimilar or non-comparable biologics, the manufacturer of the insulin), which may influence the insulin-related and total treatment cost. We also did not investigate the preference for and persistence of either modality of injecting insulin.

## Conclusion

According to this study, insulin pens are simpler, more convenient, and safe but more expensive than vial syringes. Glycemic control is comparable between pen and syringe users. Long-term follow-up studies are needed to determine the clinical and economic impacts of such benefits of insulin pens in our settings.

## Data Availability

The data used to support this study are available from the corresponding author upon request (rangassmc@gmail.com).
